# The Differential Diagnosis of Smallpox

**Published:** 1919-05-17

**Authors:** 


### THE DIFFERENTIAL DIAGNOSIS OF SMALLPOX.

In spite of the recent controversies following upon Dr. McVail's excellent lectures upon "Half a Century of Smallpox" and the discussion as to the relative degree of protection with which our counter-measures in the past have provided the present population of Great Britain, one can at least say, whichever side of the argument we support, that there is a definite possibility of local outbreaks of this disease making their appearance during the months ahead. Granted that the virulence has in recent years been reduced by an extraordinary extent and control become correspondingly easier; but, as Osler points out, even in epidemics of the milder variety, suddenly a typically malignant case will crop up. Also, if we trace back the earlier records, it is found that in the seventeenth and eighteenth centuries, before the introduction of vaccination, epidemics of the mild variety probably occurred intercurrently with the severe. We know smallpox can be one of the most fatal of contagious diseases, and that a high proportion of unvaccinated people are attacked if exposed to infection. Therefore, realising the possibility, however good our public health organisation, of a virulent strain entering and developing in this country, one feels that urgent importance may at any time arise for the immediate recognition of first cases, and it is with the object of reminding doctors in the United Kingdom, many of whom have never seen a smallpox case, of the essentials in diagnosis that these few notes are written.

### Variola Vera.

The incubation period is, on an average, twelve days, with a four-day variation in either direction, and during this time it is unusual for patients to complain of any symptoms. Then, with a series of chills in adults or a convulsion in children, intense frontal headache, severe pains in the back, and vomiting set in. The temperature rises rapidly to 103° or so. According to authorities the pains in trunk and limbs are so marked that, when combined with vomiting, they may at once suggest the nature of the disease. Within forty-eight hours initial rashes may appear and take either a scarlatinal or macular form. Occasionally they may have a general distribution, but are usually limited to the lower part of the abdomen, inner aspect of thighs, or lateral thoracic region.

Such is, roughly, the clinical picture up to the third day. On the fourth day, as generally stated, the true eruption makes its appearance, and with it the temperature falls. First on the forehead, face, and scalp there are slightly raised red papules, which give a local "shotty" feeling to the skin. Next they appear on the back of the wrists, then the trunk and arms, and, lastly, the legs, passing as a wave over the body, from above downwards. On any given area inspection will show spots of approximately the same age and development, which are circular, two to three millimetres in diameter, of relatively uniform size, bright red in colour, and which disappear completely on pressure. Although some vesiculation in these spots occurs about two days after their first appearance, it is not until the third, generally the fourth, that the process is complete. As the rash becomes vesicular pyrexia returns.

### Diagnostic Difficulties.

Unfortunately it is not a case of differentiating typical *varicella* from typical *variola*. Neither is likely to be confused. Difficulty lies in the possible aberrance of chickenpox, and, to a far greater extent, in the aberrance of smallpox resulting from a partially exhausted vaccination immunity. Past history of infection with either disease, and details of inoculation, are of paramount importance in diagnosis, but there are a number of other useful points to be noticed. The initial fever in chickenpox is usually trivial and frequently unnoticed; if present, it *increases* when the rash first appears. In smallpox there is marked initial fever, and the varioloid type generally shows an acute febrile onset; it is never so slight as to pass unnoticed. Prodromal rashes are rare in varicella, but are common and typical in distribution even in mild forms of variola.

In distribution the varicella rash gives more pocks on the trunk than on the face and limbs. The papules vesicate so rapidly that the process is usually complete within forty-eight hours of the initial appearance, but at the same time successive crops may appear over the whole body, and also many papules do not vesicate, but grow smaller and disappear. Thus in varicella pocks in all stages of development may be seen in the same skin area. In variola, the distribution has been described above, but in varioloid it is often aberrant, and, to make matters worse, a proportion of spots may abort or develop imperfectly, producing apparent difference in age in any one area. There are, however, definite points of distinction between the appearances of typical spots in most stages of the two diseases. The relative rapidity of vesiculation in varicella has been noted. The typical vesicle is here thin-walled, tense, easily ruptured and emptied by puncture, far less uniform in size, sometimes on a papular base, but often seemingly on normal skin. In variola we have slower action even in abortive infections, the vesicles are deeply embedded, thick-walled, flattened, and not easily broken. The chickenpox vesicles usually dry up within two or three days, and at their worst contain only turbid fluid. Only a few are ever truly pustular, and marked confluence of the profuse rash is not usual. In smallpox, nearly all the pocks *pustulate*, but the change is not complete until the sixth or seventh day after the rash has appeared. In instances of profuse rash, confluence is a marked characteristic.

### Comparison with Other Infections.

In the early stages, influenza or typhus may be suspected. With a prodromal rash measles is often diagnosed, but in variola there will be no initial catarrh or Koplick spots. Against scarlet fever there are several points. The severe backache, the absence of sore throat, marked prostration, normal cervical glands, and the rash may not be truly scarlatinal. One must exclude the typhus rash, ptomaine rash, drug eruptions, and many others; but if, in meeting with any illness which we ought to suspect of being smallpox, ten cardinal points were always borne in mind, and each one mentally applied to the particular case in hand, the practitioner, when considering the total of evidence so collected, should not have great difficulty in making his decision.

We have, in a typical case of variola, (1) Severe initial stage lasting three days with sudden onset and high fever, (2) backache, (3) prodromal rashes, (4) striking remission of fever as the rash first comes out, (5) shotty papules first on the forehead, (6) round, flattened, dimpled vesicles and pustules, (7) second febrile period, (8) relative distribution of rash reverse to varicella, (9) slower rate of pustule development, oldest pocks on forehead, (10) scarring and liability to confluent distribution.

